# Compound Comminuted Fracture into the Ankle Joint; Irrigation with Alcoholic Solution of Coal Tar; Rapid Recovery

**Published:** 1878-12-20

**Authors:** 


### COMPOUND COMMINUTED FRACTURE INTO THE ANKLE JOINT; IRRIGATION WITH ALCOHOLIC SOLUTION OF COAL TAR; RAPID RECOVERY.

---

Samuel M——, aged twenty-four, of unsteady habits, whilst riding, in a state of intoxication, on the shaft of a trolley loaded with two hundred weight of piping, was thrown upon the ground in consequence of the wheel rolling off the kerb-stone. The left wheel passed over his right foot from heel to ankle. On admission, (March 30, 1878), there was a longitudinal wound two inches in length in front of the ankle, opening into the joint, from which blood was oozing pretty freely. The ligaments in front of the joint were torn across, so that the articular surfaces, bare and comminuted, could be felt through the wound. The fibula was fractured a littls above the malleolus, and the tibia was obliquely fractured, the articular surfaces being to a great extent separated and comminuted, although the inner malleolus remained continuous with the shaft and its ligamentous attachments to the bones of the foot were intact. There was no displacement of the foot. Irrigation was employed in the following manner:

The limb having been laid on its outer side, flexed at the knee and kept in position by sand bags, a gutter-percha dish, with a spout for the purpose of carrying off the liquid, was placed beneath the foot, and a tin containing the fluid for irrigation was hung above the limb. The fluid employed was an alcoholic solution of coal-tar, one part of the solution to sixty of water. The "drip" action was secured by several worsted threads tied to a piece of metal at the bottom of the tin, and brought over the edge so as to hang directly over the wound. A piece of linen, wet with the lotion, was laid upon the wound, with its ends cut so as to carry off the lotion into the dish beneath the foot.

On the second day after the injury a dusky redness of the skin, with œdema, extended up to the knee, and the foot was very much swollen and inflamed; but the edges of the wound looked quiet, and its surface was covered with coagulable lymp and unaltered blood. The morning temperature was 99 degrees, the evening 100·2 degrees, and the patient said he was comfortable.

The inflammatory œdema of the leg completely subsided in four days, and that of the foot in a week. The temperature on the fifth day was 100·8 deg., on the sixth 99·4 deg., and after that it varied from 99 deg. to 88·4 deg. The pulse never rose above 58. In fact, there was no constitutional disturbance.

The coagulable lymph which filled up the gaping wound and averted its edges remained hard and glazed until the seventh day, when granulations began to sprout from its surface, and were soon covered with laudable pus.

On the seventh day a slough below the external malleolus was incised, and on the twenty-first day a large slough of the injured skin separated below the internal malleolus. On 19th of April (three weeks after the accident) the drip was discontinued, and the wounds, now quite superficial and covered with healthy granulations, were dressed with resin ointment. On the 4th of May he became an out-patient, and had then some power of flexing and extending the ankle, which promised a full and free action of the joint in no long time. *London Lancet.*

